# Combined Supplementation with Glycine and Tryptophan Reduces Purine-Induced Serum Uric Acid Elevation by Accelerating Urinary Uric Acid Excretion: A Randomized, Single-Blind, Placebo-Controlled, Crossover Study

**DOI:** 10.3390/nu11112562

**Published:** 2019-10-23

**Authors:** Shunji Oshima, Sachie Shiiya, Yasunori Nakamura

**Affiliations:** Core Technology Laboratories, Asahi Quality & Innovations, Ltd., 1-21, Midori 1-Chome, Moriya-Shi 302-0106, Japan; sachie.siiya@asahi-qi.co.jp (S.S.); yasunori.nakamura@asahi-qi.co.jp (Y.N.)

**Keywords:** glycine, purines, tryptophan, uric acid, urinary pH, urate clearance

## Abstract

The authors previously confirmed the serum uric acid-lowering effects of the combination of glycine and tryptophan in subjects with mild hyperuricemia. This study examined whether combined supplementation with glycine and tryptophan suppressed the elevation in serum uric acid levels caused by purine ingestion and accelerated urinary uric acid excretion in subjects with lower urate excretion using a randomized, single-blind, placebo-controlled, crossover clinical trial design. Healthy Japanese adult males with lower urate excretion ingested water containing purines in addition to dextrin (placebo), tryptophan, glycine, or a glycine and tryptophan mixture. The combined supplementation with glycine and tryptophan significantly reduced the elevated serum uric acid levels after purine ingestion. Glycine alone and in combination with tryptophan significantly increased urinary uric acid excretion and urate clearance compared with the effects of the placebo. Urinary pH increased by the ingestion of the mixture. These results suggested that the improved water solubility of uric acid due to increased urinary pH contributed to the increase of urinary uric acid excretion.

## 1. Introduction

The prevalence of gout, a disease of urate crystal deposition characterized by symptomatic hyperuricemia, has been increasing globally [1,2,3,4]. Gout is associated with an impaired quality of life [5,6,7] because patients present with a variety of clinical symptoms including urinary stones, acute arthritis, chronic kidney disease and metabolic syndrome [8]. The progression of gout is suggested to result from an imbalance between uric acid synthesis and excretion. The most important factor considered to increase the risk of gout is hyperuricemia with persistently high serum uric acid levels. [9]. In particular, hyperuricemia has been suggested to be caused primarily by weakened kidney excretion of uric acid [10].

Glycine, a non-essential amino acid, was revealed to enhance the urinary excretion of uric acid in healthy subjects as well as in patients with gout in previous studies [11,12,13]. Although these reports suggested that serum uric acid levels in hyperuricemia could be managed with continual glycine administration, our preliminary studies hinted that higher glycine levels were needed for effective dosing. Therefore, this study aimed to identify other amino acids that might augment the effects of glycine on serum uric acid levels. In our preliminary studies in healthy individuals, transient declines of serum uric acid levels following the oral administration of glycine together with tryptophan were observed. The uric acid-lowering effects of tryptophan, an essential amino acid, are not well known. Therefore, the authors subsequently conducted a clinical trial to confirm the serum uric acid-lowering effects of continual combined supplementation with glycine and tryptophan under mild hyperuricemia. The results revealed that combined treatment with 3.0 g of glycine and 0.2 g of tryptophan daily for 6 weeks significantly decreased serum uric acid concentrations in subjects with mild hyperuricemia [14]. The mixture of amino acids enhanced urate clearance, except in subjects with abnormally high urate clearance. The current study examined whether the combined administration of 6.0 g of glycine and 0.4 g of tryptophan suppressed the elevation in serum uric acid levels induced by purine ingestion and increased urinary uric acid excretion.

## 2. Materials and Methods

### 2.1. Volunteers

This clinical trial was conducted in accordance with the Declaration of Helsinki and the protocol was approved by the Suda Clinic Institutional Review Board (approval number: 2019-008). The volunteers were recruited from the male employees who were working in the Asahi Group Research and Development Center (Ibaraki, Japan). Written informed consent was obtained from all participating volunteers. The inclusion criteria for the study were as follows: Healthy Japanese males aged 20–64 years with lower urinary uric acid excretion (uric acid excretion <0.48 mg/kg/h according to the guidelines for the management of hyperuricemia and gout in Japan [15]) and the provision of written informed consent prior to participation. The volunteers with a history of liver, renal, heart, or severe disease, drug or food allergy and routine use of drug or dietary supplements for hyperuricemia were excluded from the study. A power analysis performed during the study planning phase indicated that more than 15 subjects needed to be recruited to achieve a study power of 0.8 at a significance level of 0.05. Thus, 16 of 43 subjects who underwent screening were enrolled according to the aforementioned inclusion and exclusion criteria.

### 2.2. Test Samples

In the study, four test samples were used. Commercially available glycine, l-tryptophan, dextrin, and disodium 5′-ribonucleotide (Ribotide^®^) were purchased from Yuki Gosei Kogyo (Tokyo, Japan), Ajinomoto Healthy Supply (Tokyo, Japan), Matsutani Chemical Industry (Tokyo, Japan) and Mitsubishi Corporation Life Sciences (Tokyo, Japan), respectively. Dextrin was used as the placebo. Disodium 5′-ribonucleotide was added as a purine source to achieve transient increases in serum uric acid levels. All volunteers ingested four test drinks (A; placebo, B; tryptophan, C; glycine, or D; glycine + tryptophan) in the crossover design and the drinks were prepared using the components listed in Table 1. All drinks were supplemented with small amounts of lemon flavor and citric acid to mask their taste and prevent the subjects from distinguishing them.

### 2.3. Study Design

**Figure 1:**
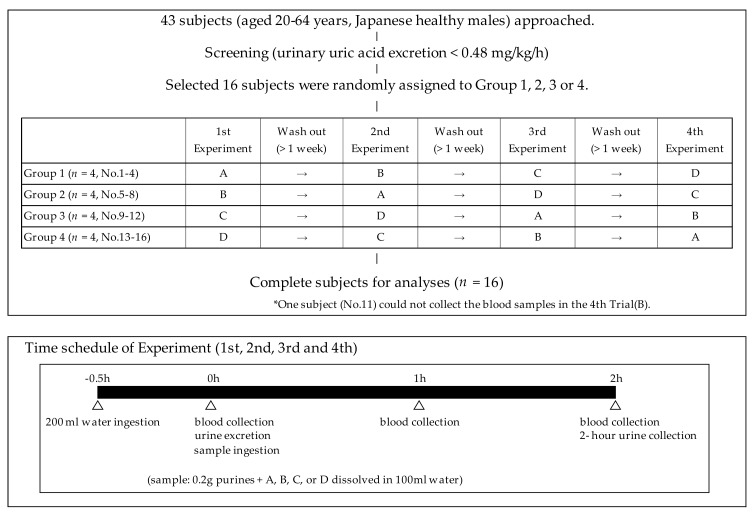
Study schedule showing the randomized, single-blind, placebo-controlled, crossover study design.

The randomized, single-blind, placebo-controlled, crossover study design was registered with the University Hospital Medical Information Network (UMIN-CTR ID: UMIN000036345, registration date: 1 April 2019) as shown in Figure 1. Sixteen healthy male volunteers were randomly allocated to group 1, 2, 3, or 4, with each group comprising four subjects. All groups received all drinks in a random order in the sequence summarized in Figure 1, with a washout period of more than 1 week between each experiment. During the study period throughout the four experiments, all subjects were instructed to maintain daily eating and drinking habits. The time schedule of the experiments is presented in Figure 1. All subjects refrained from eating and drinking alcohol for at least 10 h before the experiments. Briefly, at the beginning of the experiment, the subjects ingested 200 mL of water quickly after an overnight fast. Thirty minutes later, voided urine was excreted completely, peripheral blood was collected from the cubital vein and 100 mL of the sample drink (A, B, C, or D) was ingested. The blood was collected 1 and 2 h after the sample drink ingestion. In addition, 2-h cumulative urine specimens were collected and the urine volumes were recorded.

### 2.4. Blood and Urine Analyses

Serum uric acid, creatinine, blood urea nitrogen, albumin, total protein, alanine aminotransferase, aspartic aminotransferase, gamma-glutamyl transferase, total bilirubin, glucose, triglyceride, urinary uric acid, and creatinine levels were measured using a Fuji Drychem 7000 system (Fujifilm Co., Tokyo, Japan). Urinary pH was measured using a pH meter (D-71; Horiba, Kyoto, Japan). The urate and creatinine clearances were calculated using the following formula according to Du Bois et al. [16]: urate (or creatinine) clearance (mL/min) = urinary urate (or creatinine) excretion (mg/min)/serum urate (or creatinine) concentration (mg/mL) × 1.73 (m^3^)/body surface area (m^3^). Serum and urinary glycine and tryptophan concentrations were analyzed using an automated precolumn derivatization amino acid analytical method based on high-performance liquid chromatography/electrospray ionization mass spectrometry (UF-Amino Station system; Shimadzu, Kyoto, Japan), as previously described [17].

### 2.5. Statistical Analysis

The statistical analyses were conducted using BellCurve 2.15 software (SSRI, Tokyo, Japan). The data were presented as the mean ± standard deviation. The temporal changes at 1 and 2 h compared to pre-ingestion were analyzed for each variable using Bonferroni’s test following a repeated-measures analysis of variance and were considered significant at *p* < 0.05. The differences among the four groups were analyzed using the Tukey–Kramer test. The Pearson’s simple linear regression analysis was used to evaluate the correlations of urinary pH and urine and serum amino acid levels with urinary uric acid excretion. The differences in serum glycine/creatinine (mg/mg) between the glycine and glycine + tryptophan groups were analyzed using the sign test and the data were presented as boxplots using the medians with interquartile ranges.

## 3. Results

The characteristics of the subjects are shown in Table 2. The final analyses were performed using the data from 16 healthy male subjects aged 38.3 ± 9.2 years with lower urinary uric acid excretion levels (<0.48 mg/kg/h). The average serum uric acid concentration of the 16 subjects was 6.7 ± 0.9 mg/dL. The blood samples during the fourth experiment of the crossover trial could not be collected from one subject (No. 11) who was assigned to group 3. Therefore, all serum parameters of group 3 were analyzed with *n* = 15.

The transient changes in the serum uric acid concentrations induced by purine ingestion are summarized in Table 3. Briefly, the serum uric acid levels were significantly higher at 1 h after ingestion than at the baseline (0 h) in all groups and the serum uric acid levels remained significantly higher at 2 h versus the baseline in the placebo and tryptophan groups. Conversely, the serum uric acid levels at 2 h were comparable to the baseline levels in the glycine and glycine + tryptophan groups. The change in the serum uric acid levels from the baseline at 1 h in the glycine + tryptophan group (0.15 ± 0.15 mg/dL) was significantly smaller than in the placebo group (0.30 ± 0.17 mg/dL, *p* = 0.020). However, the change in the serum uric acid levels at 1 h was not significantly lower in the glycine (0.19 ± 0.11 mg/dL) or tryptophan groups (0.26 ± 0.13 mg/dL) than in the placebo group. The change in serum uric acid levels at 2 h versus the baseline was significantly smaller in both the glycine (0.05 ± 0.14 mg/dL) and glycine + tryptophan groups (0.01 ± 0.21 mg/dL) than in the placebo group (0.23 ± 0.11 mg/dL, *p* = 0.017 and 0.002, respectively). There was no difference in the serum uric acid levels between the tryptophan group and the other three groups. Moreover, an additional analysis was conducted in which one subject whose average serum uric acids levels exceeded 8.0 mg/dL was excluded because only patients with serum uric acid levels lower than 8.0 mg/dL should be treated with uric acid-lowering drugs according to diagnostic criteria of hyperuricemia [8]. Consequently, the change in the serum uric acid levels from the baseline to 1 h was significantly smaller in the glycine + tryptophan group (0.14 ± 0.15 mg/dL) than in the placebo group (0.28 ± 0.16 mg/dL, *p* = 0.034). The change in the serum uric acid levels at 2 h from the baseline was significantly smaller in both the glycine (0.03 ± 0.13 mg/dL) and glycine + tryptophan groups (0.02 ± 0.21 mg/dL) than in the placebo group (0.22 ± 0.11 mg/dL, *p* = 0.010 and 0.005, respectively).

The data on 2-h urinary uric acid excretion, creatinine excretion, urate clearance, creatinine clearance, and urinary pH are presented in Table 4. Briefly, urinary uric acid excretion and urate clearance were significantly higher in the glycine (0.596 ± 0.129 mg/kg/h and 9.9 ± 2.6 mL/min, respectively) and glycine + tryptophan groups (0.653 ± 0.153 mg/kg/h and 11.0 ± 2.5 mL/min, respectively) than in the placebo (0.441 ± 0.079 mg/kg/h and 7.5 ± 1.5 mL/min, respectively) and tryptophan groups (0.453 ± 0.092 mg/kg/h and 7.7 ± 1.9 mL/min, respectively). Conversely, urinary uric acid excretion and urate clearance were not significantly different between the placebo and tryptophan groups. Urinary excretion and creatinine clearance were significantly higher in the glycine group (1.43 ± 0.33 mg/kg/h and 187 ± 39 mL/min, respectively) than in the placebo (1.15 ± 0.19 mg/kg/h and 150 ± 15 mL/min, respectively) and glycine + tryptophan groups (1.16 ± 0.26 mg/kg/h and 156 ± 29 mL/min, respectively), but not the tryptophan group (1.26 ± 0.22 mg/kg/h and 176 ± 41 mL/min, respectively). The urinary excretion of uric acid per creatinine (uric acid/creatinine) was higher in the glycine + tryptophan group (0.58 ± 0.16 mg/mg) than in the other three groups (0.40 ± 0.11 mg/mg in the placebo group, 0.37 ± 0.10 mg/mg in the tryptophan group, and 0.44 ± 0.13 mg/mg in the glycine groups). Urinary pH was higher in the glycine + tryptophan group (6.83 ± 0.35) than in the placebo (6.22 ± 0.44) and tryptophan groups (6.23 ± 0.55). Although urinary pH was higher in the glycine group (6.54 ± 0.64) than in the placebo, this difference was not statistically significant. Finally, urinary pH was comparable between the tryptophan and placebo groups.

**Figure 2:**
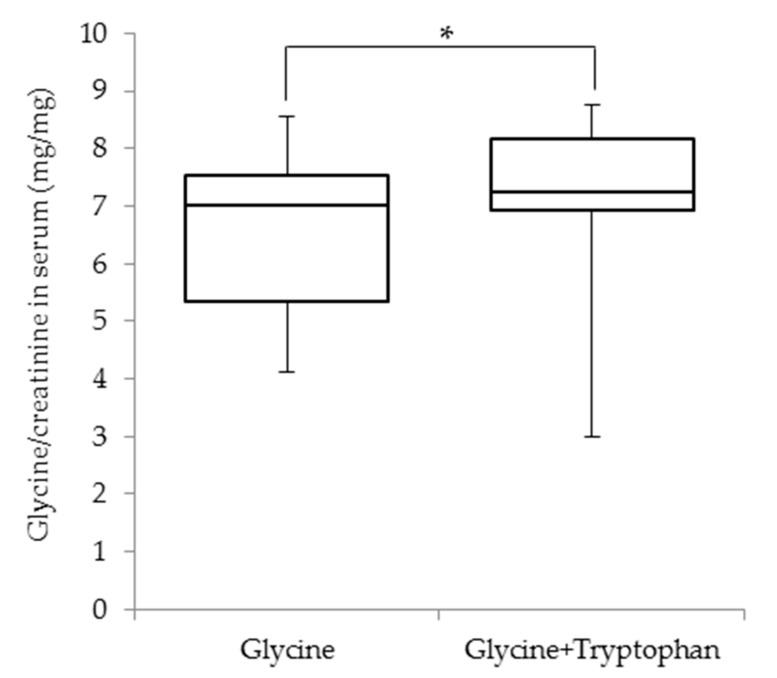
Comparison of the serum glycine/creatinine ratio in the glycine and glycine + tryptophan groups. The ratios of serum glycine/serum creatinine concentrations measured at 1 h after ingestion of the test drink are presented as boxplots (medians with interquartile ranges). * *p* < 0.05 by the sign test (*n* = 16).

The serum glycine, tryptophan, and creatinine concentrations as well as the urinary excretion of glycine and tryptophan for each group are summarized in Table 5. Briefly, the serum glycine concentrations following the test drink ingestion peaked 1 h after ingestion and the serum glycine concentrations were significantly higher at 1 and 2 h than those before ingestion in the glycine (5.7 ± 1.4 and 2.6 ± 0.4 mg/dL, respectively, versus 1.3 ± 0.2 mg/dL) and glycine + tryptophan groups (6.0 ± 1.6 and 2.6 ± 0.5 mg/dL, respectively, versus 1.3 ± 0.2 mg/dL) groups. The serum glycine concentrations did not change following the test drink ingestion in the placebo and tryptophan groups. Conversely, there was a significant increase in serum tryptophan concentrations in the tryptophan and glycine + tryptophan groups, both of which received tryptophan. Conversely, the serum creatinine levels at 2 h in the placebo group (0.85 ± 0.09 mg/dL) and at 1 and 2 h in the tryptophan (0.81 ± 0.10 and 0.83 ± 0.10 mg/dL, respectively) and glycine + tryptophan groups (0.83 ± 0.11 and 0.84 ± 0.11 mg/dL, respectively) were significant lower than those before ingestion (0.89 ± 0.11, 0.88 ± 0.10, and 0.88 ± 0.11 mg/dL in the placebo, tryptophan, and glycine + tryptophan groups, respectively). However, the serum creatinine levels did not exhibit temporal changes in the glycine group (0.88 ± 0.10, 0.87 ± 0.10, and 0.86 ± 0.10 mg/dL at 0, 1, and 2 h, respectively). Additionally, the serum glycine/creatinine ratio was significantly higher in the glycine + tryptophan group (7.3 ± 1.2 mg/mg) than in the glycine group (7.0 ± 2.2 mg/mg) at 1 h after ingestion (Figure 2). The urinary glycine excretion levels in the glycine (0.37 ± 0.23 mg/kg/h) and glycine + tryptophan (0.49 ± 0.35 mg/kg/h) groups significantly increased compared with those in the placebo (0.04 ± 0.02 mg/kg/h) and tryptophan groups (0.04 ± 0.02 mg/kg/h). Moreover, the urinary tryptophan excretion in the tryptophan (0.014 ± 0.006 mg/kg/h) and glycine + tryptophan (0.015 ± 0.005 mg/kg/h) groups significantly increased compared with those in the placebo (0.007 ± 0.003 mg/kg/h) and tryptophan groups (0.007 ± 0.003 mg/kg/h).

**Figure 3:**
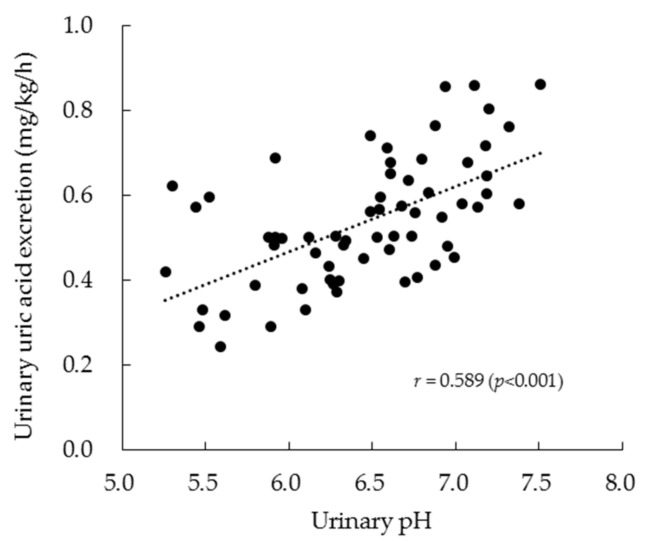
Relationship between urinary pH and urinary uric acid excretion in the crossover trial. The results represent the values (*n* = 64) from all data points of all four experiments and the correlation coefficient (*r*).

The relationship between urinary pH and urinary uric acid excretion is shown in Figure 3. The two parameters exhibited a significant positive relationship (*r* = 0.589, *p* < 0.001) based on the analysis of all data points across the four experiments. Moreover, the relationships of serum glycine and tryptophan concentrations with urinary pH and uric acid excretion were examined (Table 6). Briefly, the serum glycine levels at 1 and 2 h following ingestion of the test drinks exhibited a significant relationship with both urinary uric acid excretion and urinary pH. However, the serum tryptophan levels did not exhibit a significant relationship with either parameter.

## 4. Discussion

This study examined whether the combined administration of 6.0 g of glycine and 0.4 g of tryptophan suppressed the elevation in serum uric acid levels induced by purine ingestion and increased urinary uric acid excretion. The amino acid doses used for single ingestion were based on those used in continual supplementation [14]. Both glycine and tryptophan are present in dietary proteins that are ingested in normal daily living. However, glycine at doses of up to 90 g per day administered over several weeks were reported no serious adverse effects [18]. The oral administration of up to 5.0 g of l-tryptophan per day did not lead to any adverse effects in young adult females [19]. Therefore, no adverse effects were attributed to the combined administration of glycine and tryptophan in the current trial. This study confirmed the safety of combined glycine and tryptophan supplementation.

The current study demonstrated that the combined administration of glycine and tryptophan significantly suppressed the elevation in the serum uric acid levels caused by purine ingestion in healthy subjects. The effect of the combined amino acid administration was stronger than that of the same glycine dose alone, indicating that a small amount of tryptophan provides an additional benefit to glycine alone in reducing the elevation in the serum uric acid levels after the ingestion of dietary purines.

In agreement with previous reports [11,12], glycine increased urinary uric acid excretion and enhanced urate clearance in the current study. The authors previously reported that continual supplementation with glycine and tryptophan led to an increase in urinary pH [14]. Glycine exhibits a buffering action and it is often used as an antacid. The water solubility of uric acid increases with increasing pH levels [20] and uric acid excretion is more favorable in alkaline urine than in acidic urine [21]. Therefore, it is possible that the elevation in urinary pH caused by glycine and tryptophan supplementation enhanced the solubility of urinary uric acid, thereby elevating urinary uric acid excretion and urate clearance. Previous studies provide physiological, physicochemical and clinical validation for the use of citrate salt in the treatment of some types of lithiasis [22,23]. Citrate salts treatment substantially increased urinary pH in patients with uric acid lithiasis, however, there was no significant change in the amount of urinary uric acid [24]. The mixture of amino acids might be superior to citrate salts in the prevention or treatment of hyperuricemia. Herein, although the elevation of urinary pH induced by the ingestion of glycine alone was not significant compared with the effects of placebo, glycine administration enhanced uric acid excretion and urate clearance, raising the possibility of other mechanisms. Urate transporter 1 (URAT1), a primary reabsorptive urate transporter that is targeted by pyrazinamide [25], reportedly disables glycine-induced uricosuria [26]. Pyrazinamide may reflect the enhanced urate reabsorption following the exchange of its active metabolite [27]. Thus, glycine may inhibit the reabsorptive action of uric acid, which is induced by URAT1. Further studies are needed to elucidate the action of glycine on URAT1.

In the current study, the supplementation with tryptophan alone had no influence on urinary pH, urinary uric acid excretion or urate clearance. As the effect of tryptophan on URAT1 might be structurally difficult, tryptophan might have exerted an indirect action against uric acid elevation via its influence on the action of glycine. Specifically, tryptophan might promote the direct action of glycine against uric acid elevation. The maximum serum levels of glycine and tryptophan are achieved within an hour of ingestion, which is followed by rapid decreases as the amino acids are metabolized. Glycine metabolizes various end-products, namely glutathione, nucleic acid bases, heme, creatine and bile acids [28]. The increases in urinary creatinine excretion and creatinine clearance following the glycine challenge suggest that glycine might be partially absorbed back and metabolized to creatinine through creatine. Conversely, these urinary parameters did not change in response to the simultaneous supplementation with glycine and tryptophan, suggesting the suppression of the conversion of glycine to creatinine by tryptophan. In fact, the increases in the serum glycine/creatinine ratio and urinary glycine excretion were more pronounced in response to supplementation with glycine and tryptophan than with glycine alone. Further studies are required to clarify the mechanism underlying the contribution of tryptophan to the effects of glycine on uric acid excretion.

Among the limitations in the current clinical study, the modest sample size (*n* = 16) might have reduced the statistical power and increased the risk of type II error, particularly regarding the statistical differences in multiple comparisons between the glycine and glycine + tryptophan groups. Furthermore, the current study included only male subjects because of the known effects of sex differences on serum uric acid levels. Further studies are therefore needed, although our preliminary findings suggested that there were no effective differences between males and females in response to glycine and tryptophan supplementation (data not shown).

## 5. Conclusions

The current randomized, single-blind, placebo-controlled, crossover clinical study revealed that the combined supplementation with glycine and tryptophan significantly reduced the elevation in serum uric acid levels induced by purine ingestion via the acceleration of uric acid excretion and urate clearance in healthy males with lower urate excretion. These results suggest that improved water solubility of uric acid in response to urinary pH elevation might have contributed to the increase of urinary uric acid excretion. Tryptophan alone did not induce serum uric acid elevation or urinary excretion of uric acid, but it might have enhanced the action of glycine by regulating the metabolism of glycine to creatinine. The detailed mechanisms underlying the potential therapeutic or preventive effect of combined glycine and tryptophan supplementation should be elucidated in future studies.

## 6. Patents

Patents related to the study are as follows: Japanese Patent No. 6492236.

## Figures and Tables

**Table 1:** Components of the test drinks.

	A	B	C	D
Components	Placebo	Tryptophan	Glycine	Glycine+Tryptophan
Glycine (g)	-	-	6.0	6.0
L-Tryptophan (g)	-	0.4	-	0.4
Dextrin (g)	6.4	6.0	0.4	-
Purines (g)	0.2	0.2	0.2	0.2
Water (ml)	100	100	100	100

**Table 2:** Characteristics of the healthy male volunteers (*n* = 16).

Parameters		Means (SD)
Age (years)		38.3 (9.2)
Body weight (kg)		74.8 (10.3)
Body mass index (kg/m^2^)		25.2 (3.9)
Drinking habits		
	Drinker	14
	Non-Drinker	2
Smoking habits		
	Smoker	2
	Non-Smoker	12
	Ex-Smoker	2
Serum concentrations		
	Uric acid (mg/dL)	6.7 (0.9)
	Creatinine (mg/dL)	0.89 (0.10)
	BUN (mg/dL)	15 (2)
	Albumin (g/dL)	4.9 (0.2)
	Total protein (g/dL)	7.4 (0.3)
	ALT (U/L)	27 (12)
	AST (U/L)	24 (5)
	GGT (U/L)	27 (17)
	Total bilirubin (mg/dL)	0.7 (0.3)
	Glucose (mg/dL)	91 (6)
	Triglyceride (mg/dL)	84 (44)
	Total cholesterol (mg/dL)	190 (32)

Data are presented as the mean ± standard deviation. Abbreviations: BUN, blood urea nitrogen; AST, aspartic aminotransferase; ALT, alanine aminotransferase; GGT, gamma-glutamyl transferase.

**Table 3:** Changes of serum uric acids concentrations (mg/dL) in the crossover trial.

				Change in Uric Acid Levels	
	0 h	1 h	2 h	1 h	2 h
Placebo, *n* = 16	6.7 (1.1)	7.0 (1.2) **	6.9 (1.1) **	0.30 (0.17) ^a^	0.23 (0.11) ^a^
Tryptophan, *n* = 15	6.7 (0.9)	7.0 (0.9) **	6.9 (0.9) **	0.26 (0.13) ^a,b^	0.15 (0.17) ^a,b^
Glycine, *n* = 16	6.9 (1.0)	7.1 (1.1) **	6.9 (1.1)	0.19 (0.11) ^a,b^	0.05 (0.14) ^b^
Glycine + Tryptophan, *n* = 16	6.7 (0.9)	6.9 (0.9) **	6.7 (0.9)	0.15 (0.15) ^b^	0.01 (0.21) ^b^

Data are presented as the mean ± standard deviation. ** *p* < 0.01 by Bonferroni’s test (versus 0 h) following repeated-measures analysis of variance to assess temporal changes in each group. Mean changes in uric acid levels with different superscript letters are significantly different among the four groups at *p* < 0.05 by the Tukey–Kramer test.

**Table 4:** Comparison of urinary parameters in the placebo and amino acid supplementation groups.

	Placebo	Tryptophan	Glycine	Glycine + Tryptophan
Urinary uric acid excretion (mg/kg/h)	0.441 (0.079) ^a^	0.453 (0.092) ^a^	0.596 (0.129) ^b^	0.653 (0.153) ^b^
Urinary creatinine excretion (mg/kg/h)	1.15 (0.19) ^a^	1.26 (0.22) ^a,b^	1.43 (0.33) ^b^	1.16 (0.26) ^a^
Urinary uric acid/creatinine (mg/mg)	0.40 (0.11) ^a^	0.37 (0.10) ^a^	0.44 (0.13) ^a^	0.58 (0.16) ^b^
Urate clearance (mL/min)	7.5 (1.5) ^a^	7.7 (1.9) ^a^	9.9 (2.6) ^b^	11.0 (2.5) ^b^
Creatinine clearance (mL/min)	150 (15) ^a^	176 (41) ^a,b^	187 (39) ^b^	156 (29) ^a^
Urinary pH	6.22 (0.44) ^a^	6.23 (0.55) ^a^	6.54 (0.64) ^a,b^	6.83 (0.35) ^b^

Data are presented as the mean ± standard deviation. Means of urinary parameters with different superscript letters are significantly different among the four groups at *p* < 0.05 by the Tukey–Kramer test. Urinary uric acid and creatinine excretion and urate and creatinine clearance were analyzed in urine specimens that were collected for 2 h after complete urine excretion following ingestion of the supplement.

**Table 5:** Comparison of serum glycine and tryptophan concentrations and urinary glycine and tryptophan excretion among the placebo and amino acid groups.

	Placebo			Tryptophan			Glycine			Glycine + Tryptophan		
	0 h	1 h	2 h	0 h	1 h	2 h	0 h	1 h	2 h	0 h	1 h	2 h
Serum glycine (mg/dL)	1.2 (0.2)	1.2 (0.2)	1.2 (0.2)	1.3 (0.2)	1.2 (0.2)	1.2 (0.2)	1.3 (0.2)	5.7 (1.4) **	2.6 (0.4) **	1.3 (0.2)	6.0 (1.6) **	2.6 (0.5) **
Serum L-tryptophan (mg/dL)	1.1 (0.2)	1.1 (0.2)	1.0 (0.2)	1.1 (0.1)	2.4 (0.3) **	1.8 (0.3) **	1.1 (0.1)	1.1 (0.2)	1.1 (0.2)	1.0 (0.1)	2.2 (0.4) **	1.7 (0.2) **
Serum creatinine (mg/dL)	0.89 (0.11)	0.86 (0.09)	0.85 (0.09) *	0.88 (0.10)	0.81 (0.10) **	0.83 (0.10) *	0.88 (0.10)	0.87 (0.10)	0.86 (0.10)	0.88 (0.11)	0.83 (0.11) **	0.84 (0.11) **
Urinary glycine excretion (mg/kg/h)		0.04 (0.02) ^a^			0.04 (0.02) ^a^			0.37 (0.23) ^b^			0.49 (0.35) ^b^	
Urinary tryptophan excretion (mg/kg/h)		0.007 (0.003) ^a^			0.014 (0.006) ^b^			0.007 (0.003) ^a^			0.015 (0.005) ^b^	

Data are presented as the mean ± standard deviation. ** *p* < 0.01 by Bonferroni’s test (versus 0 h) following repeated-measures analysis of variance for temporal changes in the serum levels of each parameter. Means of urinary parameters with different superscript letters are significantly different among the four groups at *p* < 0.05 by the Tukey–Kramer test. Urinary amino acid excretion levels were analyzed in urine specimens that were collected for 2 h after complete urine excretion following ingestion of the supplement.

**Table 6:** Relationship of urinary uric acid excretion or pH with serum amino acids levels.

	Serum Glycine Levels			Serum Tryptophan Levels		
	0 h	1 h	2 h	0 h	1 h	2 h
Urinary uric acid excretion	−0.010	0.560 **	0.546 **	−0.126	0.046	0.017
Urinary pH	−0.006	0.385 **	0.419 **	−0.147	0.087	0.052

Data are presented as correlation coefficients to explore the relationship of serum amino acid levels with urinary uric acid excretion and urinary pH (*n* = 63, ** *p* < 0.01).

## References

[1] Rai S.K., Aviña-Zubieta J.A., McCormick N., de Vera M.A., Shojania K., Sayre E.C., Choi H.K. (2017). The rising prevalence and incidence of gout in British Columbia, Canada: Population-based trends from 2000 to 2012. Semin. Arthritis Rheum..

[2] Kuo C.F., Grainge M.J., Mallen C., Zhang W., Doherty M. (2015). Rising burden of gout in the UK but continuing suboptimal management: A nationwide population study. Ann. Rheum. Dis..

[3] Winnard D., Wright C., Taylor W.J., Jackson G., Te Karu L., Gow P.J., Arroll B., Thornley S., Gribben B., Dalbeth N. (2012). National prevalence of gout derived from administrative health data in Aotearoa New Zealand. Rheumatology.

[4] Zhu Y., Pandya B.J., Choi H.K. (2011). Prevalence of gout and hyperuricemia in the US general population: The National Health and Nutrition Examination Survey 2007-2008. Arthritis Rheum..

[5] Roddy E., Zhang W., Doherty M. (2007). Is gout associated with reduced quality of life? A case-control study. Rheumatology.

[6] Singh J.A., Strand V. (2008). Gout is associated with more comorbidities, poorer health-related quality of life and higher healthcare utilisation in US veterans. Ann. Rheum. Dis..

[7] Lee S.J., Hirsch J.D., Terkeltaub R., Khanna D., Singh J.A., Sarkin A., Kavanaugh A. (2009). Perceptions of disease and health-related quality of life among patients with gout. Rheumatology.

[8] Yamanaka H. (2011). Japanese guideline for the management of hyperuricemia and gout: Second edition. Nucleos. Nucleot. Nucl..

[9] Brook R.A., Forsythe A., Smeeding J.E., Lawrence E.N. (2010). Chronic gout: Epidemiology, disease progression, treatment and disease burden. Curr. Med. Res. Opin..

[10] Anzai N., Endou H. (2007). Drug discovery for hyperuricemia. Expert Opin. Drug Discov..

[11] Christman A.A., Mosier E.C. (1929). Purine metabolism. II. The effect of the ingestion of glycine on the excretion of endogenous uric acid. J. Biol. Chem..

[12] Friedman M. (1947). The effect of glycine on the production and excretion of uric acid. J. Clin. Investig..

[13] Kersley G.D., Mandel L., Bene E. (1951). Gout: Observations on the effects of drugs on plasma uric acid and urinary uric acid. Ann. Rheum. Dis..

[14] Oshima S., Shiiya S., Nakamura Y. (2019). Serum Uric acid-lowering effects of combined glycine and tryptophan treatments in subjects with mild hyperuricemia: A randomized, double-blind, placebo-controlled, crossover study. Nutrients.

[15] Shio H. (2012). Characteristics of the guideline for the management of hyperuricemia and gout, 2nd edition. Health Eval. Promot..

[16] Du Bois D., Du Bois E.F. (1916). Clinical calorimetry: Tenth paper a formula to estimate the approximate surface area if height and weight be known. Arch. Intern. Med..

[17] Yoshida H., Kondo K., Yamamoto H., Kageyama N., Ozawa S., Shimbo K., Muramatsu T., Imaizumi A., Mizukoshi T., Masuda J. (2015). Validation of an analytical method for human plasma free amino acids by high-performance liquid chromatography ionization mass spectrometry using automated precolumn derivatization. J. Chromatogr. B Analyt. Technol. Biomed. Life Sci..

[18] Pérez-Torres I., Zuniga-Munoz A.M., Guarner-Lans V. (2017). Beneficial effects of the amino acid glycine. Mini-Rev. Med. Chem..

[19] Hiratsuka C., Fukuwatari T., Sano M., Saito K., Sasaki S., Shibata K. (2013). Supplementing healthy women with up to 5.0 g/d of L-tryptophan has no adverse effects. J. Nutr..

[20] Wilcox W.R., Khalaf A., Weinberger A., Kippen I., Klinenberg J.R. (1972). Solubility of uric acid and monosodium urate. Med. Biol. Eng..

[21] Kanbara A., Miura Y., Hyogo H., Chayama K., Seyama I. (2012). Effect of urine pH changed by dietary intervention on uric acid clearance mechanism of pH-dependent excretion of urinary uric acid. Nutr. J..

[22] Pak C.Y.C., Fuller C., Sakhaee K., Preminger G.M., Britton F. (1985). Long-term treatment of calcium nephrolithiasis with potassium citrate. J. Urol..

[23] Alvarez M.A., Traba M.L., Rapado A. (1992). Hypocitraturia as a pathogenic risk factor in the mixed (calcium oxalate/uric acid) renal stones. Urol. Int..

[24] Sakhaee K., Nicar M., Hill K., Pak C.Y., Sakhaee K. (1983). Contrasting effects of potassium citrate and sodium citrate therapies on urinary chemistries and crystallization of stone-forming salts. Kidney Int..

[25] Ichida K., Hosoyamada M., Hisatome I., Enomoto A., Hikita M., Endou H., Hosoya T. (2004). Clinical and molecular analysis of patients with renal hypouricemia in Japan-influence of URAT1 gene on urinary urate excretion. J. Am. Soc. Nephrol..

[26] Kaung C., Gutman A.B. (1970). Effect of glycine loading on plasma and urinary uric acid and amino acids in normal and gouty subjects. Am. J. Med..

[27] Roch-Ramel F., Guisan B., Schild L. (1996). Indirect coupling of urate and p-aminohippurate transport to sodium in human brush-border membrane vesicles. Am. J. Physiol..

[28] Reeds P.J., Mersmann H.J. (1991). Protein and energy requirements of animals treated with β-adrenergic agonists: A discussion. J. Anim. Sci..

